# Association of State COVID-19 Vaccine Mandates With Staff Vaccination Coverage and Staffing Shortages in US Nursing Homes

**DOI:** 10.1001/jamahealthforum.2022.2363

**Published:** 2022-07-29

**Authors:** Brian E. McGarry, Ashvin D. Gandhi, Maggie Syme, Sarah D. Berry, Elizabeth M. White, David C. Grabowski

**Affiliations:** 1 Division of Geriatrics and Aging, Department of Medicine, University of Rochester Medical Center, Rochester, New York; 2 Anderson School of Management, University of California Los Angeles; 3 Hinda and Arthur Marcus Institute for Aging Research, Hebrew SeniorLife, Boston, Massachusetts; 4 Beth Israel Deaconess Medical Center, Department of Medicine, Harvard Medical School, Boston, Massachusetts; 5 Department of Health Services, Policy & Practice, Brown University School of Public Health, Providence, Rhode Island; 6 Department of Health Care Policy, Harvard Medical School, Boston, Massachusetts

## Abstract

**Importance:**

Several states implemented COVID-19 vaccine mandates for nursing home employees, which may have improved vaccine coverage but may have had the unintended consequence of staff departures.

**Objective:**

To assess whether state vaccine mandates for US nursing home employees are associated with staff vaccination rates and reported staff shortages.

**Design, Setting, and Participants:**

This cohort study performed event study analyses using National Healthcare Safety Network data from June 6, 2021, through November 14, 2021. Changes in weekly staff vaccination rates and reported staffing shortages were evaluated for nursing homes in states with mandates after the mandate announcement compared with changes in facilities in nonmandate states. An interaction between the mandates and county political leaning was considered. Data analysis was performed from February to March 2022.

**Exposures:**

Weeks after announcement of a state’s COVID-19 vaccine mandate.

**Main Outcomes and Measures:**

Weekly percentage of all health care staff at a nursing home who received at least 1 COVID-19 vaccine dose, and a weekly indicator of whether a nursing home reported a staffing shortage.

**Results:**

Among 38 study-eligible states, 26 had no COVID-19 vaccine mandate for nursing home employees, 4 had a mandate with a test-out option, and 8 had a mandate with no test-out option. Ten weeks or more after mandate announcement, nursing homes in states with a mandate and no test-out option experienced a 6.9 percentage point (pp) increase in staff vaccination coverage (95% CI, −0.1 to 13.9); nursing homes in mandate states with a test-out option experienced a 3.1 pp increase (95% CI, 0.5 to 5.7) compared with facilities in nonmandate states. No significant increases were detected in the frequency of reported staffing shortages after a mandate announcement in mandate states with or without test-out options. Increases in vaccination rates in states with mandates were larger in Republican-leaning counties (14.3 pp if no test-out option; 4.3 pp with option), and there was no evidence of increased staffing shortages.

**Conclusions and Relevance:**

The findings of this cohort study suggest that state-level vaccine mandates were associated with increased staff vaccination coverage without increases in reported staffing shortages. Vaccination increases were largest when mandates had no test-out option and were also larger in Republican-leaning counties, which had lower mean baseline vaccination rates. These findings support the use of state mandates for booster doses for nursing home employees because they may improve vaccine coverage, even in areas with greater vaccine hesitancy.

## Key Points

**Question:**

Are state COVID-19 vaccine mandates for US nursing home employees associated with staff vaccination coverage and reported staff shortages?

**Findings:**

This cohort study of nursing homes in 38 states found that states with a vaccine mandate experienced an increase in staff vaccination coverage compared with facilities in states with no mandate and no worsening of reported staffing shortages following the mandates.

**Meaning:**

These findings suggest that given the waning vaccine-induced immunity and low booster dose coverage among nursing home staff in many parts of the US, state mandates for booster doses may be warranted to improve and sustain vaccination coverage in nursing homes.

## Teaser

This cohort study of nursing homes in 38 US states examines how state COVID-19 vaccine mandates and test-out options affected staff vaccination coverage and staffing shortages in Republican- and Democratic-leaning counties.

## Introduction

High COVID-19 vaccination coverage among direct care staff is critical to avoid and manage nursing home COVID-19 outbreaks and deaths,^1,2^ yet many staff remained unvaccinated months after vaccines became available.^3^ COVID-19 vaccination for nursing home staff serves 3 purposes: (1) to protect residents who are particularly vulnerable to severe infection and may not mount sufficient vaccine-induced immunity,^4^ (2) to protect staff who themselves have experienced a high toll of infection and morbidity,^5,6^ and (3) to control viral transmission to mitigate nursing home outbreaks.^1,7^ New COVID-19 variants along with low staff vaccination rates in many US nursing homes despite extensive coordinated vaccination campaigns have led several states to implement mandates requiring nursing home employees to be vaccinated against COVID-19.^8,9^ These state policies were mostly introduced ahead of the federal mandate that was announced as a final rule by the Centers for Medicare & Medicaid Services (CMS) on November 4, 2021.^10^

How state COVID-19 vaccine mandates affected nursing home workers is largely unknown. Specifically, it is not clear whether mandates necessarily increased staff vaccination rates, and there are several reasons for this lack of clarity. First, state mandates may include a number of potential exemptions that might allow many workers to remain unvaccinated. A number of states adopted mandates that had a test-out option for employees who wanted to remain unvaccinated for any reason, meaning that staff could choose to submit to additional COVID-19 testing in lieu of receiving the vaccine. Likewise, state policies may allow for medical or religious exemptions from the mandate. Finally, mandates may not have been strictly enforced, thereby allowing out-of-compliance staff to continue working.

How state COVID-19 vaccine mandates affected the supply of nursing home workers is also unclear. Many nursing home leaders have expressed substantial concerns that requiring vaccination as a prerequisite for working in a nursing home may lead many direct care staff to leave the industry, potentially worsening the already severe worker shortages experienced by nursing homes throughout the pandemic.^11,12^

Lastly, how the success of state COVID-19 vaccine mandates may be affected by the predominant political preferences of a given geographic location is also unknown. This factor can strongly influence perceptions of vaccines,^13^ and has been shown to correlate strongly with vaccine acceptance and uptake.^3,14,15^ Consequently, mandates may have varying success in terms of achieving their intended policy goal (eg, increasing staff vaccination coverage without worsening staff shortages) in areas with differing political leanings.

A study in Mississippi evaluated how the nation’s first state-level COVID-19 vaccine mandate affected nursing home employees and staff vaccine coverage.^16^ The findings indicated that in the weeks after the June 15, 2021, enactment , the nursing home staff vaccination rates increased compared with comparator states without a mandate; however, the gains were modest and failed to raise levels to industry benchmarks. Notably, Mississippi’s policy provided a test-out option for employees.

The purpose of this cohort study was to examine the association of state COVID-19 vaccine mandates with staff vaccination coverage and staffing shortages at nursing homes. We examined these relationships among states with and without test-out options in their mandates and across counties with different political leanings.

## Methods

Consistent with Harvard Medical School policy for publicly available data, this study was exempt from formal institutional review. Study design and reporting of results was informed by the Strengthening the Reporting of Observational Studies in Epidemiology (STROBE) reporting guidelines.

### Data Source

Outcomes for this study were measured from the CMS COVID-19 Nursing Home Public File, a publicly available resource that includes data from all Medicare- and Medicaid-certified nursing homes submitted weekly via the US Centers for Disease Control and Prevention’s National Healthcare Safety Network Long-term Care Facility COVID-19 Module.^17^ The module covers a number of topics, including weekly counts of COVID-19 cases and deaths among staff and residents. The weekly survey also includes questions about staff vaccination rates and whether the facility experienced a staff shortage in the prior week. Details on outcome construction using these questions are provided in the following section.

Data on state COVID-19 vaccine mandate policies were collected from a number of sources, including internet searches using Google, state websites, state memos, and news reports. We collected information on whether a state had a mandate that applied to nursing home employees, the date on which the mandate was announced, the date on which the mandate went into effect, and whether the state allowed nursing home employees to submit to additional testing in lieu of vaccination (ie, a test-out option). We compiled information for mandates enacted from June 15, 2021, to November 30, 2021.

### Outcomes and Variable Construction

#### Staff Vaccination Rates

The primary outcome was the weekly percentage of all health care staff who were eligible to work for 1 day or more (including staff on temporary leave) during the data collection week who received at least 1 dose of an authorized COVID-19 vaccine (Moderna, Pfizer-BioNTech, or Janssen).

#### Staff Shortage Rates

Staffing shortages were determined weekly at the facility level according to whether a nursing home reported a shortage in any of the following categories: nurses (registered nurses and licensed practical nurses), clinical staff (physicians, physician assistants, and advanced practice nurses), aides (certified nursing assistants, nurse aides, and medication aides or technicians), and other (ie, staff not involved in direct resident care [eg, food or environmental service staff]). Reported state-level shortage rates were the estimated number of nursing homes in a state that had reported a shortage during a given week divided by the total number of sample nursing homes in that state.

#### County Political Leaning

County-level 2020 US Presidential election results were obtained from publicly available sources.^18^ Republican-leaning counties were defined as counties with greater than 50% of the vote share going to the Republican presidential candidate. Democratic-leaning counties were defined as those with 50% or less of the vote share going to the Republican candidate.

#### Exposure

To allow for anticipatory effects, we defined exposure as the week in which the state mandate was announced. In a sensitivity analysis, we defined separate exposures for mandate announcement and mandate enactment. Additionally, we created separate categories for mandates with and without a test-out option.

### Sample

Sample nursing homes included those located in states where we could identify the mandate status and announcement date. States with mandates that applied to only a subgroup of nursing home workers (eg, state employees) were excluded. We also excluded nursing homes in Mississippi because that state had announced a mandate before reporting of staff vaccination rates was required in the National Healthcare Safety Network data (ie, we lacked premandate outcome data for this state). Lastly, we excluded nursing homes in Alaska because its presidential election results were not reported at the county level.

Weekly outcomes were measured from June 6, 2021, to November 14, 2021. This limited study window was selected to avoid confounding from the announcement of the federal mandate that was announced on November 4, 2021, with an initial targeted enactment date of December 6, 2021.^19^

### Statistical Analysis

To determine the association of state mandates with staff vaccination coverage and staffing shortages, we estimated an event study model that characterizes the change in the outcome of interest in mandate states after the mandate announcement compared with the changes experienced in nonmandate states during the same time period. Our main estimates of interest in this approach were indicators for each week in relation to mandate announcement (ie, weeks before mandate announcement, <0; week of the announcement, = 0; weeks after announcement week, >0) for states with a mandate. Nonmandate states served as a comparison group; these event-time indicators were 0 in all periods for nonmandate states. We estimated effects for event-times before the mandate as a means by which to test the plausibility of our method’s assumption: that mandate and nonmandate states would follow similar trends in the absence of a mandate.

Event study models were estimated using linear regressions that included fixed effects for each nursing home and calendar week. We clustered standard errors at the state level because mandates were determined at the state level.

To quantify the magnitude of estimated effects, we used fitted regression models to estimate what staff vaccination and staff shortage rates would have been in states with a mandate, with or without a test-out option, had they never announced or implemented a mandate. We graphically compared these projected values with the actual mean vaccination and staff shortage rates observed for states with a mandate, with and without a test-out option, over calendar time.

A similar event study approach was used to separately examine the association of mandates in Republican-leaning and Democratic-leaning counties. We compared changes in the outcomes of interest in Republican- or Democratic-leaning counties in mandate states after the mandate announcement with the changes experienced in Republican- or Democratic-leaning counties in nonmandate states during the same time period using interactions between weeks-relative-to-mandate-announcement estimates and an indicator for whether each nursing home was located in a Republican-leaning county. Models also included separate calendar week fixed effects for Republican- and Democratic-leaning counties. Additional details are available in eAppendix 1 in the Supplement.

Statistical tests were 2-tailed and *P* values < .05 were considered statistically significant. Data analyses were performed from February to March 2022 using Stata, version 16 (StataCorp LLC).

### Sensitivity Analyses

We performed a number of sensitivity analyses to test the robustness of the results against alternate specifications. We estimated mandate effects using a difference-in-differences (DID) model with fixed effects for facility and calendar week. Within this DID framework, we also estimated separate effects for mandate announcement and enactment. Similarly for analyses examining the association of mandates with vaccine coverage and shortage rates in Republican- and Democratic-leaning counties, we used a difference-in-difference-in-differences (DDD) model that formally tested whether the estimated effect of mandates differed by county-level political leaning within each state. Furthermore, we used the DDD framework to test the sensitivity of these results against more stringent thresholds for defining Republican-leaning counties (eg, Republican vote share ≥60%, ≥70%, and ≥80%). Additional details are available in eTable 1 and eAppendix 1 in the Supplement.

## Results

**Figure 1.**
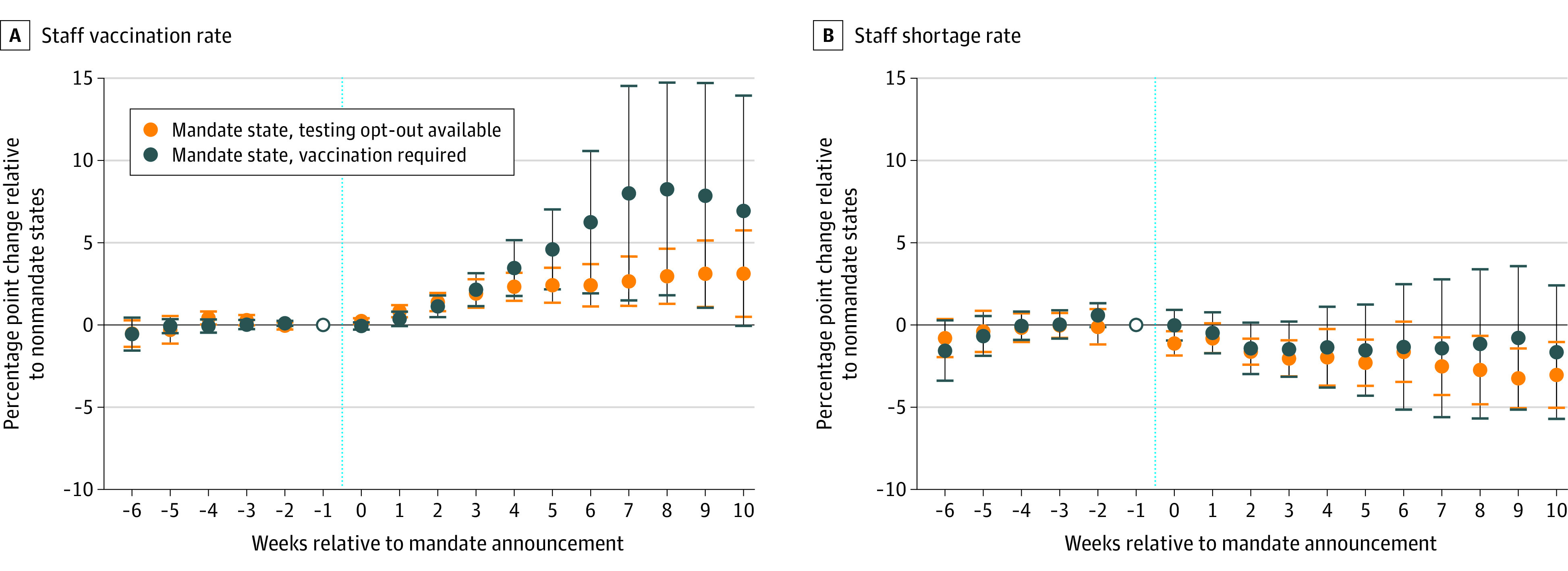
Event Study Estimates of the Association of COVID-19 Vaccine Mandates With Nursing Home Staff Vaccination Coverage and Reported Staff Shortages Estimated weekly differences and 95% CIs in staff vaccination coverage (A) and reported staff shortage rates (B) between mandate states (with and without a test-out option) and nonmandate states after a mandate announcement (denoted by the vertical dashed line). Estimates were obtained from an event study regression with facility and calendar week fixed effects. All estimates are relative to the week prior to mandate announcement (week = −1), denoted by an open circle in the figure. Full details are available in eAppendix 2 in the Supplement. Event week = −6 contains estimates for event weeks ≤ −6. Event week = 10 contains estimates for event weeks ≥ 10.

**Figure 2.**
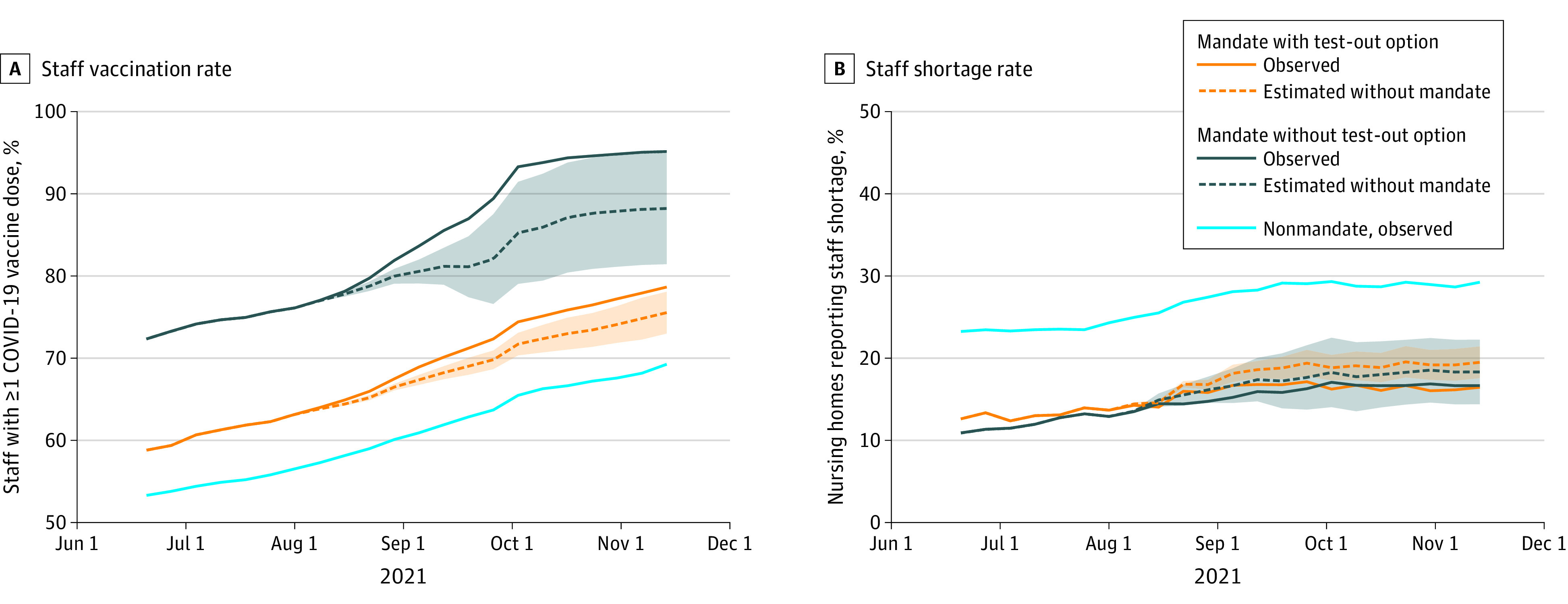
Nursing Home Staff Vaccination Coverage and Reported Staffing Shortages by Mandate Type, Observed and Projected If Mandate Had Not Been Adopted Figure plots observed (solid line) and projected (dashed line; in the absence of state mandates) coverages of nursing home staff with at least 1 dose of a COVID-19 vaccine (A) and the percent of nursing homes reporting a staff shortage (B) in states with vaccine mandates (with and without a test-out option). Projected estimates were obtained from event study regression estimates presented in Figure 1. All estimates are relative to the week prior to mandate announcement (week = −1), denoted by an open circle in the figure. Shaded areas represent 95% CIs of projected values.

Among 38 states with available data, 26 had no mandate, 4 had a mandate with a test-out option, and 8 had a mandate with no test-out option (Table). Ten weeks or longer after the mandate announcement, nursing homes in states with a mandate and no test-out option experienced a 6.9 percentage point (pp) increase in staff vaccination coverage (95% CI, −0.1 to 13.9), while nursing homes in mandate states with a test-out option experienced a 3.1 pp increase (95% CI, 0.5 to 5.7) compared with facilities in states with no mandate (Figure 1). By November 14, 2021, this equated to mean vaccination coverage of 95.2% and 78.7% in mandate states without and with a test-out option, respectively, compared with predicted vaccination coverage of 88.3% (95% CI, 81.5% to 95.0%) and 75.6% (95% CI, 73.1% to 78.1%) had those mandates not been adopted (Figure 2). Nonmandate states had consistently lower staff vaccination coverage throughout the study window. We detected no significant increases in the frequency of reported staff shortages following mandate announcement in either mandate states with or without a test-out option. Nonmandate states had higher rates of reported staff shortages throughout the study period (Figure 2).

**Table.** Characteristics of Study-Eligible Mandate States

State	Mandate type	Date of announcement	Date of enactment
California	No test out	August 5, 2021	September 30, 2021
Colorado	No test out	August 17, 2021	November 30, 2021
Connecticut	No test out	August 6, 2021	September 7, 2021
Delaware	Test out	August 12, 2021	September 30, 2021
Illinois	Test out	August 26, 2021	October 4, 2021
Kentucky	Test out	August 3, 2021	October 1, 2021
Massachusetts	No test out	August 4, 2021	October 10, 2021
New Jersey	Test out	August 6, 2021	September 6, 2021
New Mexico	No test out	August 17, 2021	August 23, 2021
New York	No test out	August 16, 2021	September 27, 2021
Oregon	No test out	August 19, 2021	October 18, 2021
Washington	No test out	August 9, 2021	October 18, 2021

Results were consistent when using a DID analytic approach and jointly comparing all weeks following mandate announcement between mandate states, with and without a test-out option, and nonmandate states. For instance, mean staff vaccine coverage was estimated to increase by 5.4 pp (95% CI, 1.1-9.8) in mandate states without a test-out option and 2.2 pp (95% CI, 0.8-3.5) in mandate states with a test-out option following mandate announcement relative to observed trends in states with no mandate (eTable 2 in the Supplement). Likewise, no evidence of increased staff shortage rates was found in this alternate specification.

**Figure 3.**
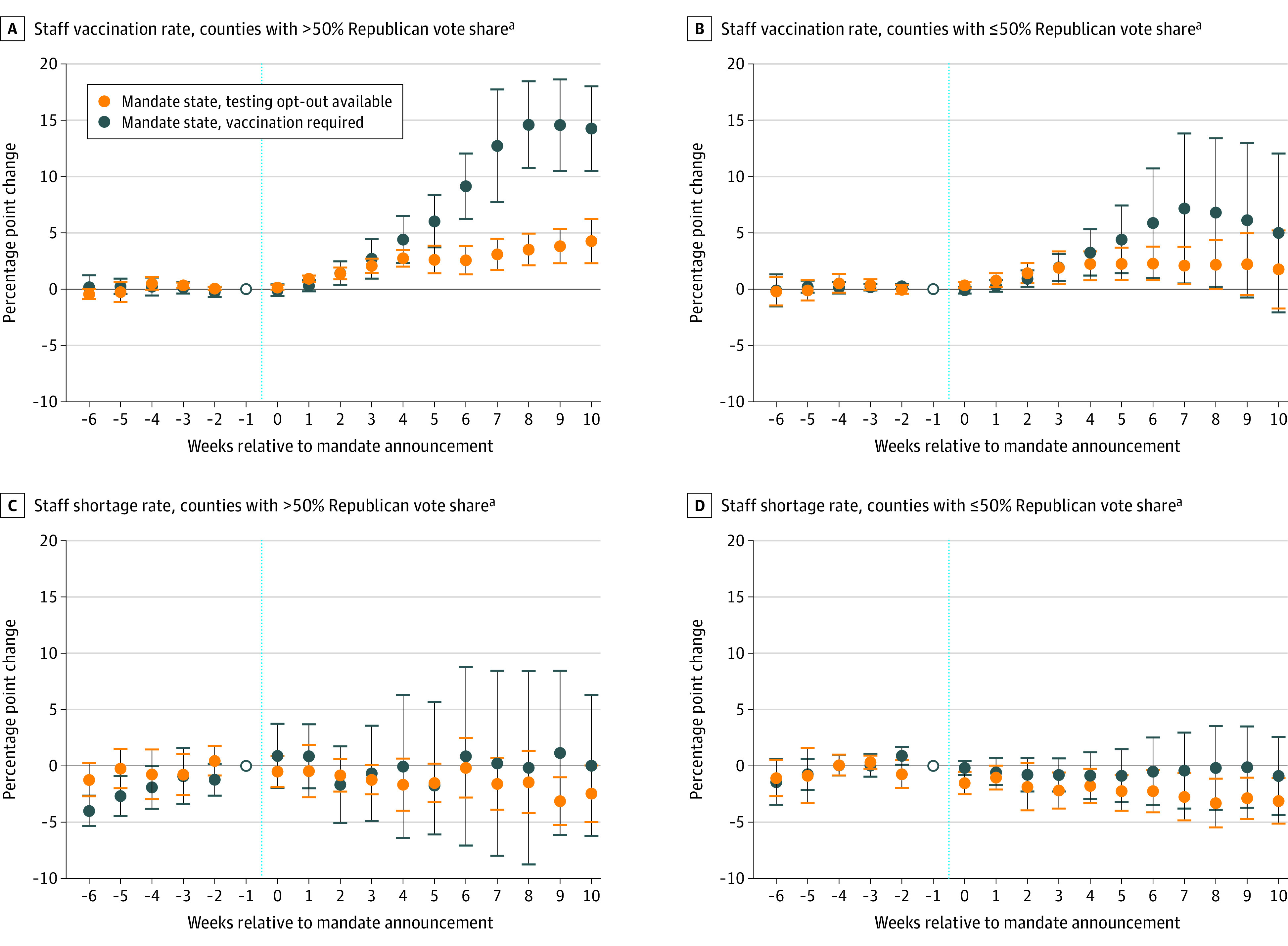
Nursing Home Staff Vaccination Coverage and Reported Staff Shortages After Mandate Announcement, by Mandate Type and County Voting Patterns ^a^Defined as the share of votes for the Republican presidential candidate in the 2020 election. Figure plots estimated weekly differences and 95% CIs in staff vaccination coverage (A and C) and reported staff shortage rates (B and D) by county political preference between mandate states (with and without a test-out option) and nonmandate states following mandate announcement (denoted by the vertical dashed line). Estimates are obtained from an event study regression with facility and separate calendar week fixed effects for Republican- and Democratic-leaning counties. Full details are available in eAppendix 2 in the Supplement. Event week = −6 contains estimates for event weeks ≤ −6. Event week = 10 contains estimates for event weeks ≥10.

Increases in mandate effects on vaccination coverages were larger in Republican-leaning counties without evidence of increased reported staff shortages. Among nursing homes in Republican-leaning counties, those in states with a mandate and no test-out option experienced a 14.3 pp increase (95% CI, 10.5-18.0) in staff vaccination coverage 10 or more weeks following announcement relative to Republican-leaning counties in nonmandate states, while those in a state with a mandate and a test-out option experienced an increase in staff vaccination coverage of 4.3 pp (95% CI, 2.3-6.2; Figure 3; eTables 3 and 4 in the Supplement). Formal comparisons of Republican-leaning and Democrat-leaning counties within the same states using a DDD approach indicated that increases were significantly larger in Republican-leaning counties (eTables 5 in the Supplement). Following mandate announcement, Republican-leaning counties experienced a 5.0 pp increase (95% 1.5-8.6) in staff vaccine coverage relative to Democrat-leaning counties in mandate states without a test-out option and a 1.5 pp increase (95% CI, 0.4-2.7) in mandate states with a test-out option. Findings were generally consistent when more restrictive thresholds for defining a Republican-leaning county were used (eTable 6 in the Supplement).

## Discussion

State-level vaccine mandates were associated with increased staff vaccination coverage without increases in reported staff shortages. Increases in vaccination coverage were largest when mandates had no test-out option, suggesting that strict mandates may be an effective policy tool to improve staff vaccination rates. Increases in vaccination coverage in mandate states were also larger in Republican-leaning counties, which had lower mean vaccination coverage at baseline (eFigure in the Supplement), suggesting that mandates were effective in areas where there may be political opposition to such policies.

The association of mandates with higher vaccination coverage stands in contrast with prior efforts to increase COVID-19 vaccine uptake among nursing home staff through education, outreach, and incentives. For example, a cluster-randomized trial evaluating a multicomponent vaccine campaign found no significant difference in staff vaccine coverage between intervention and control facilities.^20^ This suggests that compelling staff vaccination through mandates may be the best available option to policy makers to achieve and maintain high levels of COVID-19 vaccination among this population.

Ongoing monitoring of vaccination coverage and staffing levels will be essential as nursing homes in all states become subject to the federal vaccine mandate recently upheld by the US Supreme Court and as states consider whether to mandate booster doses. As of May 8, 2022, 87.9% of nursing home employees had completed their primary vaccine series but only half (50.3%) of those vaccinated staff had received booster doses.^6^ There has been relatively little action by states to mandate booster doses for nursing home employees. One exception is Massachusetts, which required nursing home staff to receive a COVID-19 additional dose or booster vaccination by February 28, 2022. Not surprisingly, Massachusetts reports the highest share of staff in nursing homes with complete COVID-19 vaccination who have received additional primary or booster doses (96.3% as of May 16, 2022). Given the waning of vaccine-induced immunity with new variants,^21^ and evidence that higher nursing home staff coverage is associated with fewer resident infections and deaths,^1^ further state mandates for booster doses may be warranted.

The biggest concern with imposing vaccine mandates for nursing home employees is the potential for large numbers of staff to leave the workforce rather than be vaccinated. For example, when a nursing home in Philadelphia required staff to be vaccinated, 17 (6.9%) of the facility’s 246 employees chose to resign.^22^ Low nurse staffing levels have been consistently associated with poor resident outcomes in nursing homes.^23,24,25^ Even before the COVID-19 pandemic, many nursing homes reported nursing ratios below the recommended threshold.^26,27^ Staff turnover and shortages have intensified during the pandemic,^11,28^ and policy makers do not want to enact policies that exacerbate shortfalls. As a resident’s family member summarized, “You’re reluctant to do something that could cause you to lose the people you rely on.”^29^ However, the findings of this study suggest that nursing homes did not report worsened staffing shortfalls following the announcement of a state mandate. This result held even for strict mandates that did not allow for a test-opt option and for facilities in Republican-leaning counties. This finding is encouraging and suggests that the fear of massive staffing shortfalls owing to vaccine mandates may be unfounded.

### Limitations

All the data used in this study were self-reported by the nursing homes. Thus, in the context of a mandate, facilities may be incentivized to report higher vaccination rates. Nursing homes may also be reticent to report staffing shortages, particularly for direct care staff, because they could trigger deficiency citations. Furthermore, these measures may not be sensitive enough to detect staff departures. For example, a facility could experience the loss of a registered nursing owing to the mandate, but this departure might not lead administrators to report a new shortage because the facility has sufficient registered nurses, or by contrast, was already reporting a shortage. Future work will need to analyze mandates and nursing home staffing levels using more granular staff payroll data. Finally, we evaluated how mandates affected staff vaccination status and shortages beginning with the date on which the mandate was publicly announced. It is possible that certain facilities and staff members were aware of the plan to enact mandates in the weeks before the announcement, and this could have biased the results to the null. To account for these anticipatory changes, we included the week of announcement in the follow-up period.

## Conclusions

In this cohort study of nursing homes located in states that did and did not enact a COVID-19 vaccine mandate for nursing home staff, state mandates were associated with increases in staff vaccine coverage without any reported increase in staffing shortages. These findings held in Republican-leaning counties which, on average, had lower baseline vaccination rates. Moreover, we found that stricter mandates with no test-out option were associated with increased vaccine coverage relative to policies that allowed a test-out option. These findings suggest that state mandates for booster doses for nursing home employees could be associated with improved vaccine coverage, even in areas with greater vaccine hesitancy.

## Notes

Supplement. eAppendix 1. Supplementary Details on Methods eAppendix 2. Additional Results eTable 1. Event Study Regression Estimates eTable 2. Results of Difference-in-Differences Analyses eFigure. Unadjusted Mean Staff Vaccine Coverage and Staff Shortage Rates by State Mandate Status and County Political Leanings eTable 3. Event Study Regression Estimates for Staff Vaccination Coverage by County Political Leanings eTable 4. Event Study Regression Estimates for Reported Staff Shortages by County Political Leanings eTable 5. Results of Difference-in-Difference-in-Differences Analyses to Test for Differential Mandate Effects by County Political Leanings eTable 6. Results of Difference-in-Difference-in-Differences Analyses using More Restrictive Definitions of Republican leaning
