# Supplementary material for: Association of State COVID-19 Vaccine Mandates With Staff Vaccination Coverage and Staffing Shortages in US Nursing Homes
Source: JAMA Health Forum. 2022 Jul 29;3(7):e222363. doi: 10.1001/jamahealthforum.2022.2363 (PMC9338409; doi:10.1001/jamahealthforum.2022.2363)
Supplement: Supplement. — eAppendix 1. Supplementary Details on Methods eAppendix 2. Additional Results eTable 1. Event Study Regression Estimates eTable 2. Results of Difference-in-Differences Analyses eFigure. Unadjusted Mean Staff Vaccine Coverage and Staff Shortage Rates by State Mandate Status and County Political Leanings eTable 3. Event Study Regression Estimates for Staff Vaccination Coverage by County Political Leanings eTable 4. Event Study Regression Estimates for Reported Staff Shortages by County Political Leanings eTable 5. Results of Difference-in-Difference-in-Differences Analyses to Test for Differential Mandate Effects by County Political Leanings eTable 6. Results of Difference-in-Difference-in-Differences Analyses using More Restrictive Definitions of Republican leaning [file jamahealthforum-e222363-s001.pdf]

Supplemental Online Content

McGarry BE, Gandhi AD, Syme M, Berry SD, White EM, Grabowski DC. Association of state COVID-19 vaccine mandates with staff vaccination coverage and staffing shortages in US nursing homes. *JAMA Health Forum.* 2022;3(7):e222363. doi:10.1001/jamahealthforum.2022.2363

eAppendix 1. Supplementary Details on Methods

eAppendix 2. Additional Results

eTable 1. Event Study Regression Estimates

eTable 2. Results of Difference-in-Differences Analyses

eFigure. Unadjusted Mean Staff Vaccine Coverage and Staff Shortage Rates by State Mandate Status and County Political Leanings

eTable 3. Event Study Regression Estimates for Staff Vaccination Coverage by County Political Leanings

eTable 4. Event Study Regression Estimates for Reported Staff Shortages by County Political Leanings

eTable 5. Results of Difference-in-Difference-in-Differences Analyses to Test for Differential Mandate Effects by County Political Leanings

eTable 6. Results of Difference-in-Difference-in-Differences Analyses using More Restrictive Definitions of Republican leaning

This supplemental material has been provided by the authors to give readers additional information about their work.

© 2022 McGarry et al. *JAMA Health Forum.*

E-APPENDIX 1. SUPPLEMENTARY DETAILS ON METHODS

Analysis

Equation 1 summarizes our main event study analytic approach used to estimate the effect of state mandates on staff vaccination coverage and staff shortages, relative to the changes in outcomes experienced in non-mandate states over the same time period.

$$Y_{i,t} = \eta^{N}_{t-\tau(i)} M^{N}_{i} + \eta^{O}_{t-\tau(i)} M^{O}_{i} + \gamma_i + \theta_t + \epsilon_{i,t} \qquad \text{Eq. 1}$$

$Y_{i,t}$ represents the outcome of interest for facility i in calendar week t. γ_i is a facility fixed effect and θ_t represents calendar week fixed effects. M^{N}_{i} and M^{O}_{i} are indicators for whether facility i is in a mandate state with testing opt-out and without a testing opt-out, respectively. Note that both indicators will be zero for facilities in a state without a mandate. For facility i in a state with a mandate, $\tau(i)$ gives the announcement date of the mandate so that $t - \tau(i)$ gives the event time during calendar week t. (Note that we truncate event weeks at 6 weeks prior to mandate announcement—i.e., all weeks prior to 6 weeks before mandate announcement are set equal to -6—and 10 weeks following mandate announcement.) The coefficients η^{N}_{k} and η^{O}_{k} respectively represent our estimate of the effect of mandate announcement on event week k. We estimate effects for event-times prior to the mandate as well as a means by which to test the plausibility of our method's assumption: that mandate and non-mandate states would follow similar trends in absence of the mandate. We cluster our standard errors at the state level because mandates are determined at the state level.

Equation 2 summarizes the event study model used to separately examine the effects of mandates in Republican-leaning and Democratic-leaning counties. Specifically, we estimate a version of Equation 1 that includes interactions between mandate effect estimates and an indicator for nursing home i being located in a Republican-leaning County (R_i) and separate time trends for Republican- and Democratic-leaning counties.

$$Y_{i,t} = \eta^{N}_{t-\tau(i)} M^{N}_{i} + \eta^{N}_{t-\tau(i)} M^{N}_{i} R_i + \eta^{O}_{t-\tau(i)} M^{O}_{i} + \eta^{O}_{t-\tau(i)} M^{O}_{i} R_i + \gamma_i + \theta_t + \theta_t R_i + \epsilon_{i,t} \qquad \text{Eq. 2}$$

Sensitivity Analyses

To examine the robustness of our results we performed a series of sensitivity analyses. First we estimated overall mandate effects using a differences-in-differences (DID) analytic approach instead of an event study approach (Equation 3).

$$Y_{i,t} = Post^{N}_{i,t} + Post^{0}_{i,t} + \gamma_i + \theta_t + \epsilon_{i,t} \qquad \text{Eq. 3}$$

Where $Post^{N}_{i,t}$ is an indicator for all calendar weeks following mandate announcement in states without a test-out option, $Post^{N}_{i,t}$ calendar weeks following mandate announcement in states with a test-out option, and γ_i and θ_t represents facility and calendar week fixed effects respectively. $Post^{N}_{i,t}$ and $Post^{N}_{i,t}$ are the standard DID estimates of interest, representing the mean change in the outcomes of interest after mandate announcement relative the changes observed in non-mandate states over the same time period.

Second, we use the DID framework to estimates separate effects of mandate announcement and mandate enactment.

Third, we extend Equation 3 to formally examine whether estimates mandate effects differ between Republican- and Democrat-leaning counties within the same state. This is

© 2022 McGarry et al. *JAMA Health Forum.*

accomplished through use of a triple differences (DDD) model that compares relative changes in outcomes in Republican-leaning counties in mandate states (in comparison to Republican-counties in non-mandate states) to the relative changes in Democrat-leaning counties in mandate states (in comparison to Democrat-leaning counties in non-mandate states) following mandate announcement. Equation 4 summarizes this approach with R_i being an indicator for facility i being in a county with a majority vote share going to the Republican candidate in the 2020 presidential election.

$$Y_{i,t} = Post_{i,t}^{N} + Post_{i,t}^{N}R_i + Post_{i,t}^{0} + Post_{i,t}^{0}R_i + \gamma_i + \theta_t + \theta_t R_i + \epsilon_{i,t} \quad \text{Eq. 3}$$

Fourth, we test the sensitivity of our results to the threshold at which we consider a county to be Republican-leaning. We re-estimate Equation 3 when Republican counties are restricted to those with at least 60%, 70%, or 80% of vote share going to the Republican candidate.

© 2022 McGarry et al. *JAMA Health Forum.*

eAppendix 2- Additional Results

eTable 1- Event Study Regression Estimates

	Change staff vaccination coverage (pp) relative to the week prior to mandate announcement		Change in staff shortage rate (pp) relative to the week prior to mandate announcement	
Event Time (weeks relative to mandate announcement)	Test-out option available	No test-out option	Test-out option available	No test-out option
≤ -6	-0.52	-0.55	-0.80	-1.57*
	(-1.32, 0.29)	(-1.55, 0.44)	(-1.97, 0.36)	(-3.41, 0.27)
-5	-0.30	-0.07	-0.40	-0.68
	(-1.14, 0.54)	(-0.49, 0.35)	(-1.65, 0.85)	(-1.89, 0.53)
-4	0.42**	-0.07	-0.17	-0.06
	(0.03, 0.81)	(-0.46, 0.33)	(-1.04, 0.70)	(-0.92, 0.81)
-3	0.29*	0.02	-0.03	0.03
	(-0.03, 0.61)	(-0.25, 0.29)	(-0.80, 0.73)	(-0.84, 0.89)
-2	-0.04	0.10	-0.12	0.58
	(-0.24, 0.15)	(-0.05, 0.26)	(-1.19, 0.95)	(-0.15, 1.32)
-1	REF	REF	REF	REF
0	0.24***	-0.06	-1.13***	-0.03
	(0.06, 0.41)	(-0.28, 0.16)	(-1.86, -0.39)	(-0.96, 0.91)
1	0.83***	0.37	-0.82*	-0.48
	(0.46, 1.20)	(-0.08, 0.82)	(-1.72, 0.08)	(-1.74, 0.78)
2	1.39***	1.13***	-1.63***	-1.42*
	(0.83, 1.94)	(0.47, 1.79)	(-2.42, -0.83)	(-2.98, 0.14)
3	1.91***	2.14***	-2.04***	-1.48*
	(1.05, 2.77)	(1.13, 3.15)	(-3.14, -0.94)	(-3.16, 0.21)
4	2.33***	3.46***	-1.97**	-1.36
	(1.47, 3.18)	(1.76, 5.16)	(-3.69, -0.25)	(-3.81, 1.09)
5	2.42***	4.59***	-2.30***	-1.54
	(1.35, 3.48)	(2.16, 7.03)	(-3.71, -0.89)	(-4.31, 1.23)
6	2.41***	6.24***	-1.64*	-1.34
	(1.13, 3.70)	(1.91, 10.57)	(-3.47, 0.20)	(-5.15, 2.47)
7	2.65***	8.00**	-2.52***	-1.42
	(1.15, 4.15)	(1.47, 14.52)	(-4.26, -0.77)	(-5.63, 2.79)
8	2.96***	8.25**	-2.74**	-1.16
	(1.28, 4.64)	(1.80, 14.70)	(-4.82, -0.65)	(-5.68, 3.37)
9	3.11***	7.85**	-3.24***	-0.79
	(1.08, 5.14)	(1.02, 14.69)	(-5.06, -1.43)	(-5.15, 3.57)
≥ 10	3.12**	6.93*	-3.04***	-1.66
	(0.49, 5.74)	(-0.07, 13.93)	(-5.03, -1.04)	(-5.71, 2.40)
*** p<0.01, ** p<0.05, * p<0.1				

© 2022 McGarry et al. *JAMA Health Forum.*

Notes: Table presents the regression estimates and 95% confidence intervals presented in Figure 1 of the manuscript for staff vaccine coverage. Estimates were obtained from the regression summarized in Equation 1.

© 2022 McGarry et al. *JAMA Health Forum.*

eTable 2- Results of Difference-in-Differences Analyses

		Staff Vaccine Coverage (%)		Staff Shortage (%)	
		I	*II*	*III*	*IV*
**Mandate, test-out option available**	Relative change in outcome (pp) after mandate announcement	2.18***	1.71***	-1.78*	-1.13
		(0.83, 3.53)	(0.78, 2.65)	(-3.62, 0.05)	(-2.60, 0.33)
	Relative change in outcome (pp) after mandate enactment		1.13		-1.28***
			(-0.47, 2.74)		(-2.18, -0.37)
**Mandate, required**	Relative change in outcome (pp) after mandate announcement	5.43**	3.76***	-0.75	-0.55
		(1.08, 9.78)	(1.81, 5.72)	(-4.44, 2.95)	(-3.37, 2.27)
	Relative change in outcome (pp) after mandate enactment		3.96		-0.48
			(-2.27, 10.19)		(-2.98, 2.01)
95% confidence intervals in parentheses					
* p < 0.10, ** p < 0.05, *** p < 0.01					

Notes: Results obtained from a difference-in-difference models containing facility and calendar week fixed effects and indicators for facilities located in a state with a mandate (with and without a test-out option). Columns I and III present results from models with a single post-period (i.e., post mandate announcement). Columns II and IV present results from models with separate indicators for weeks that are post mandate announcement and weeks that are post mandate enactment.

© 2022 McGarry et al. *JAMA Health Forum.*

eFigure 1- Unadjusted Mean Staff Vaccine Coverage and Staff Shortage Rates by State Mandate Status and County Political Leanings

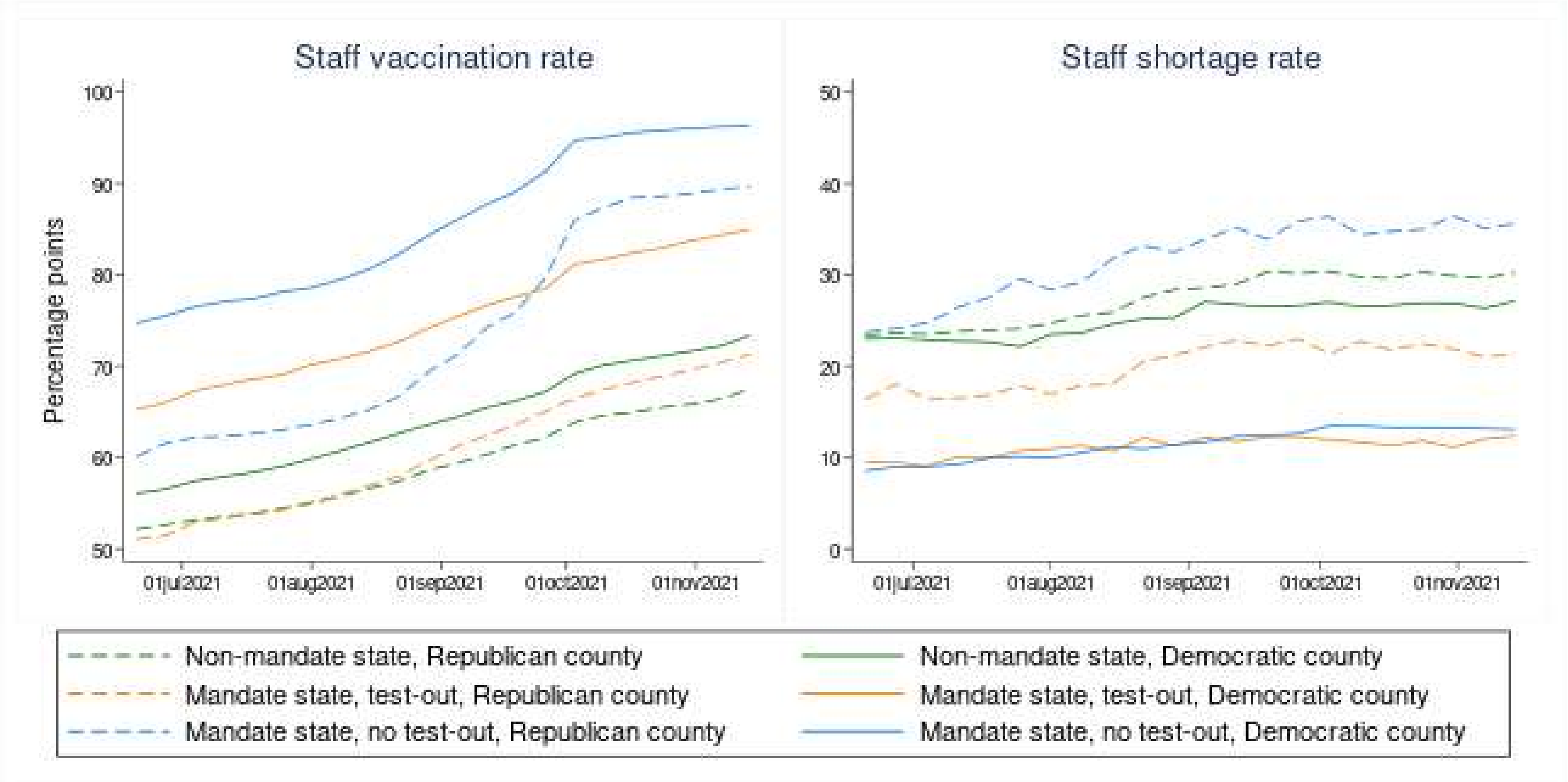

© 2022 McGarry et al. *JAMA Health Forum.*

eTable 3- Event Study Regression Estimates for Staff Vaccination Coverage by County Political Leanings

	**Change staff vaccination coverage (pp) relative to the week prior to mandate announcement**			
Even t Time	Test-out option available		No test-out option	
	Democrat-leaning County	*Republican-leaning County*	*Democrat-leaning County*	*Republican-leaning County*
≤ -6	-0.22	-0.46*	-0.12	0.16
	(-1.46, 1.03)	(-0.93, 0.02)	(-1.55, 1.31)	(-0.92, 1.23)
-5	-0.12	-0.27	0.20	0.24
	(-1.02, 0.78)	(-1.18, 0.64)	(-0.34, 0.74)	(-0.46, 0.93)
-4	0.50	0.50*	0.12	0.21
	(-0.35, 1.35)	(-0.07, 1.07)	(-0.38, 0.63)	(-0.58, 1.00)
-3	0.37	0.33***	0.16	0.12
	(-0.11, 0.84)	(0.10, 0.56)	(-0.13, 0.46)	(-0.41, 0.66)
-2	-0.06	0.04	0.24**	-0.27
	(-0.44, 0.31)	(-0.12, 0.20)	(0.041, 0.45)	(-0.72, 0.18)
-1	REF	REF	REF	REF
0	0.33***	0.14	-0.11	-0.12
	(0.09, 0.57)	(-0.08, 0.35)	(-0.39, 0.18)	(-0.61, 0.38)
1	0.76**	0.91***	0.27	0.28
	(0.12, 1.40)	(0.63, 1.19)	(-0.27, 0.81)	(-0.26, 0.82)
2	1.41***	1.38***	0.91**	1.40**
	(0.51, 2.31)	(0.84, 1.92)	(0.16, 1.66)	(0.35, 2.45)
3	1.89**	2.06***	1.92***	2.69***
	(0.44, 3.34)	(1.40, 2.71)	(0.77, 3.07)	(0.91, 4.47)
4	2.05***	2.73***	3.23***	4.39***
	(0.76, 3.33)	(2.00, 3.47)	(1.16, 5.31)	(2.31, 6.48)
5	2.23***	2.60***	4.39***	6.01***
	(0.81, 3.65)	(1.37, 3.82)	(1.36, 7.42)	(3.66, 8.35)
6	2.26***	2.55***	5.86**	9.13***
	(0.78, 3.74)	(1.28, 3.82)	(1.02, 10.71)	(6.21, 12.04)
7	2.08**	3.08***	7.15**	12.71***
	(0.44, 3.73)	(1.69, 4.47)	(0.48, 13.82)	(7.73, 17.70)
8	2.16**	3.50***	6.79**	14.59***
	(0.01, 4.32)	(2.10, 4.91)	(0.19, 13.39)	(10.73, 18.44)
9	2.20	3.80***	6.11*	14.57***
	(-0.55, 4.95)	(2.27, 5.34)	(-0.75, 12.97)	(10.51, 18.62)
≥ 10	1.75	4.25***	4.99	14.25***
	(-1.70, 5.21)	(2.29, 6.22)	(-2.07, 12.05)	(10.50, 18.00)
*** p<0.01, ** p<0.05, * p<0.1				

Notes: Table presents the regression estimates and 95% confidence intervals presented in Figure 3 of the manuscript for staff vaccine coverage. Estimates were obtained from the regression summarized in Equation 2.

© 2022 McGarry et al. *JAMA Health Forum.*

eTable 4- Event Study Regression Estimates for Reported Staff Shortages by County Political Leanings

	**Change staff shortages (pp) relative to the week prior to mandate announcement**			
Event Time	Test-out option available		No test-out option	
	Democrat-leaning County	*Republican-leaning County*	*Democrat-leaning County*	*Republican-leaning County*
≤ -6	-1.09	-1.25*	-1.45	-4.01***
	(-2.70, 0.51)	(-2.73, 0.22)	(-3.47, 0.57)	(-5.36, -2.66)
-5	-0.88	-0.25	-0.75	-2.69***
	(-3.34, 1.57)	(-1.99, 1.49)	(-2.13, 0.63)	(-4.46, -0.92)
-4	0.05	-0.77	0.036	-1.91*
	(-0.88, 0.97)	(-2.98, 1.45)	(-0.87, 0.94)	(-3.82, 0.00)
-3	0.32	-0.78	0.02	-0.93
	(-0.28, 0.91)	(-2.59, 1.03)	(-0.98, 1.02)	(-3.43, 1.57)
-2	-0.75	0.43	0.89**	-1.23*
	(-2.00, 0.51)	(-0.87, 1.74)	(0.10, 1.68)	(-2.65, 0.18)
-1	REF	REF	REF	REF
0	-1.54***	-0.51	-0.19	0.88
	(-2.53, -0.55)	(-1.89, 0.87)	(-0.80, 0.43)	(-1.99, 3.75)
1	-1.05*	-0.47	-0.58	0.85
	(-2.11, 0.01)	(-2.80, 1.86)	(-1.88, 0.71)	(-1.98, 3.68)
2	-1.87*	-0.85	-0.79	-1.68
	(-3.97, 0.23)	(-2.30, 0.61)	(-2.27, 0.69)	(-5.08, 1.72)
3	-2.19***	-1.25*	-0.81	-0.68
	(-3.80, -0.58)	(-2.55, 0.06)	(-2.29, 0.66)	(-4.90, 3.55)
4	-1.78**	-1.68	-0.87	-0.07
	(-3.30, -0.26)	(-4.02, 0.67)	(-2.93, 1.19)	(-6.41, 6.27)
5	-2.42***	-1.53*	-0.88	-0.23
	(-3.99, -0.85)	(-3.25, 0.20)	(-3.25, 1.48)	(-6.11, 5.66)
6	-2.25**	-0.18	-0.52	0.84
	(-4.13, -0.36)	(-2.84, 2.47)	(-3.54, 2.51)	(-7.07, 8.76)
7	-2.76**	-1.60	-0.42	0.21
	(-4.85, -0.67)	(-3.91, 0.70)	(-3.79, 2.95)	(-8.00, 8.42)
8	-3.31***	-1.46	-0.19	-0.17
	(-5.48, -1.15)	(-4.23, 1.30)	(-3.91, 3.53)	(-8.77, 8.42)
9	-2.87***	-3.14***	-0.13	1.14
	(-4.73, -1.01)	(-5.23, -1.04)	(-3.74, 3.49)	(-6.14, 8.42)
≥ 10	-3.13***	-2.46*	-0.90	0.01
	(-5.15, -1.11)	(-4.97, 0.04)	(-4.37, 2.56)	(-6.26, 6.29)
*** p<0.01, ** p<0.05, * p<0.1				

Notes: Table presents the regression estimates and 95% confidence intervals presented in Figure 3 of the manuscript for reported staff shortages. Estimates were obtained from the regression summarized in Equation 2.

© 2022 McGarry et al. *JAMA Health Forum.*

eTable 5- Results of Difference-in-Difference-in-Differences Analyses to Test for Differential Mandate Effects by County Political Leanings

		**Staff Vaccine Coverage (%)**	**Staff Shortage (%)**
**Mandate, test-out option available**	Change in outcome (pp) after mandate announcement in Democrat-leaning counties	1.26**	-1.75**
		(0.01, 2.52)	(-3.40, -0.11)
	Change in outcome (pp) after mandate announcement in Republican-leaning counties relative to Democrat-leaning counties	1.54***	0.85
		(0.43, 2.66)	(-1.75, 3.46)
**Mandate, no test-out option available**	Change in outcome (pp) after mandate announcement in Democrat-leaning counties	4.12*	-0.15
		(-0.31, 8.54)	(-3.11, 2.81)
	Change in outcome (pp) after mandate announcement in Republican-leaning counties relative to Democrat-leaning counties	5.04***	2.35
		(1.52, 8.55)	(-1.08, 5.78)

© 2022 McGarry et al. *JAMA Health Forum.*

eTable 6- Results of Difference-in-Difference-in-Differences Analyses using More Restrictive Definitions of Republican leaning

		Republican Leaning Vote Share					
		≥ 60%		≥ 70%		≥ 80%	
		Staff Vaccine Coverage (%)	Staff Shortage (%)	Staff Vaccine Coverage (%)	Staff Shortage (%)	Staff Vaccine Coverage (%)	Staff Shortage (%)
Mandate, test-out option available	Change in outcome (pp) after mandate announcement in Democrat-leaning counties	1.83***	-2.07**	1.89***	-2.24**	2.06***	-1.69*
		(0.63, 3.02)	(-3.77, -0.38)	(0.50, 3.27)	(-3.94, -0.54)	(0.71, 3.41)	(-3.44, 0.05)
	Change in outcome (pp) after mandate announcement in Republican-leaning counties relative to Democrat-leaning counties	0.69	1.98	1.29**	3.35**	1.81	1.89
		(-0.84, 2.23)	(-1.25, 5.22)	(0.12, 2.46)	(0.59, 6.10)	(-0.45, 4.08)	(-2.02, 5.80)
Mandate, no test-out option available	Change in outcome (pp) after mandate announcement in Democrat-leaning counties	4.90**	-0.15	5.28**	-0.34	5.34**	-0.50
		(0.59, 9.21)	(-3.59, 3.29)	(0.91, 9.66)	(-3.89, 3.21)	(0.99, 9.69)	(-4.14, 3.13)
	Change in outcome (pp) after mandate announcement in Republican-leaning counties relative to Democrat-leaning counties	5.04***	3.91*	4.13**	2.12	1.06	-5.85**
		(1.52, 8.55)	(-0.47, 8.29)	(0.08, 8.18)	(-8.37, 12.61)	(-5.95, 8.06)	(-10.77, -0.94)

© 2022 McGarry et al. *JAMA Health Forum.*
